# Albuminuria predicts kidney events in IgA nephropathy

**DOI:** 10.1093/ndt/gfae085

**Published:** 2024-04-30

**Authors:** Anne-Laure Faucon, Sigrid Lundberg, Stefania Lando, Julia Wijkström, Mårten Segelmark, Marie Evans, Juan-Jesús Carrero

**Affiliations:** Department of Medical Epidemiology and Biostatistics, Karolinska Institutet, Stockholm, Sweden; Department of Clinical Epidemiology, Centre for Research in Epidemiology and Population Health, INSERM U1018, Paris-Saclay University, Villejuif, France; Department of Medical Specialist Care, Nephrology Clinic, Danderyd University Hospital, Stockholm, Sweden; Department of Clinical Sciences, Karolinska Institutet, Danderyd Hospital, Stockholm, Sweden; MedTechLabs, BioClinicum, Karolinska University Hospital, Solna, Sweden; Department of Medical Epidemiology and Biostatistics, Karolinska Institutet, Stockholm, Sweden; University of Milano-Bicocca, Milan, Italy; Department of Nephrology, Karolinska University Hospital, Stockholm, Sweden; Department of Clinical Science, Intervention and Technology, Karolinska Institutet, Stockholm, Sweden; Department of Clinical Sciences, Lund University, Lund, Sweden; Department of Endocrinology, Nephrology and Rheumatology, Skåne University Hospital, Lund, Sweden; Department of Nephrology, Karolinska University Hospital, Stockholm, Sweden; Department of Clinical Science, Intervention and Technology, Karolinska Institutet, Stockholm, Sweden; Department of Medical Epidemiology and Biostatistics, Karolinska Institutet, Stockholm, Sweden; Department of Clinical Sciences, Karolinska Institutet, Danderyd Hospital, Stockholm, Sweden

**Keywords:** albuminuria, chronic kidney disease, IgA nephropathy, kidney replacement therapy

## Abstract

**Background and hypothesis:**

KDIGO recommends proteinuria <1 g/d as a treatment target in patients with immunoglobulin A nephropathy (IgAN) because of high risk of progression to kidney failure. However, long-term kidney outcomes in patients with low-grade proteinuria remain insufficiently studied.

**Methods:**

We enrolled patients with biopsy-proven primary IgAN from the Swedish Renal Registry and analyzed associations between urine albumin-to-creatinine ratio (uACR, in categories <0.3, 0.3–0.5, 0.5–1.0, 1.0–1.5, 1.5–2.0, and ≥2.0 g/g) and the occurrence of major adverse kidney events [MAKE, a composite of kidney replacement therapy (KRT) and >30% decline in estimated glomerular filtration rate (eGFR)]. We also explored the risk of kidney events associated with change in uACR within a year.

**Results:**

We included 1269 IgAN patients (74% men, median 53 years, mean eGFR 33 ml/min/1.73 m², median uACR 0.7 g/g). Over a median follow-up of 5.5 [2.8; 9.2] years, 667 MAKE and 517 KRT events occurred, and 528 patients experienced >30% eGFR decline. Compared with uACR < 0.3 g/g, any higher uACR category was strongly and incrementally associated with the risk of MAKE [adjusted hazard ratios (HR) ranging from 1.56 (95%CI 1.14–2.14) if uACR 0.3–0.5 g/g to 4.53 (3.36–6.11) if uACR ≥ 2.0 g/g], KRT (HR ranging from 1.39 to 4.65), and eGFR decline >30% (HR ranging from 1.76 to 3.47). In 785 patients who had repeated uACR measurements within a year, and compared with stable uACR, the risk of kidney events was lower if uACR decreased by 2-fold (HR ranging from 0.47 to 0.49), and higher if uACR increased by 2-fold (HR from 1.18 to 2.56), irrespective of baseline uACR.

**Conclusions:**

There is substantial risk of adverse kidney outcomes among patients with IgAN and uACR between 0.3 and 1.0 g/g, a population currently considered at low risk of CKD progression. Reduction in uACR is associated with better kidney outcomes, irrespective of baseline uACR.

## Graphical Abstract

**Graphical Abstract:**
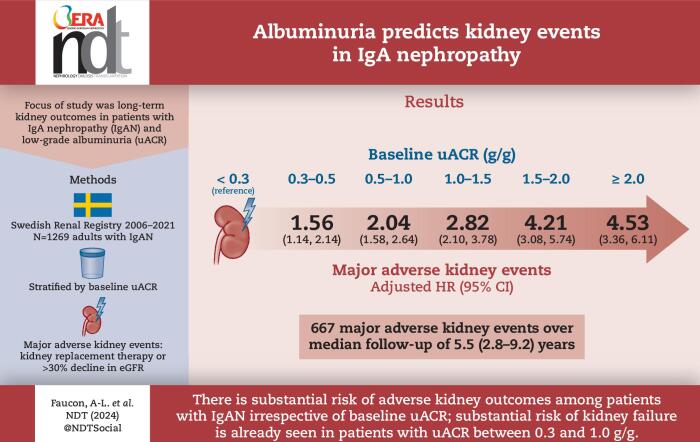

KEY LEARNING POINTS **What was known:**

- Immunoglobulin A nephropathy (IgAN) is the most common etiology of primary glomerular diseases and a major cause of kidney failure worldwide.
- Proteinuria is a widely recognized risk factor for progression to kidney failure. KDIGO recommends proteinuria <1 g/d as a treatment target in patients with IgAN because of the high risk of progression to kidney failure.
- However, whether low-grade proteinuria predicts adverse kidney outcomes in IgAN remains insufficiently studied.

**This study adds:**

- There is a strong and incremental association between urine albumin-to-creatinine ratio (uACR) and the risk of adverse kidney events.
- A substantial risk of kidney failure is already seen in patients with uACR between 0.3 and 1.0 g/g, a population currently considered at low risk of chronic kidney disease (CKD) progression.
- Any reduction in uACR, irrespective of baseline uACR, is associated with better kidney outcomes.

**Potential impact:**

- Our results inform clinical decisions for risk stratification and optimization of clinical and pharmacological management to delay the risk of CKD progression in IgAN.
- Our findings provide a rational to re-evaluate the current risk-based treatment target of proteinuria <1 g/d in IgAN.

## INTRODUCTION

Immunoglobulin A nephropathy (IgAN) is the most common etiology of primary glomerular diseases [1], and a major cause of kidney failure worldwide, particularly in young people. IgAN is an immune-complex mediated glomerulonephritis with deposition of under-galactosylated IgA in the glomerular mesangium, leading to mesangio-proliferative glomerulonephritis [2, 3]. The clinical presentation and the course of the disease are heterogenous, varying from asymptomatic microscopic hematuria and/or low-grade proteinuria to rapidly progressive glomerulonephritis. Despite substantial progress in the understanding of its pathophysiology and the emergence of new targeted treatment strategies, IgAN remains a progressive disease, with an estimated 20%–40% of patients with IgAN developing kidney failure within 10 to 20 years following diagnosis [4–6, 7].

Proteinuria is the most important and widely recognized risk factor for progression to kidney failure, and proteinuria reduction is now accepted as a surrogate for hard kidney outcomes in studies on therapeutic interventions in IgAN [8]. Observational studies have shown a strong and consistent association between the level, duration, and change in proteinuria and the risk of progression to kidney failure [8–15, 16]. Likewise, data from controlled trials have shown a direct association between treatment and reduction in proteinuria while subsequently lowering the risk of kidney outcomes [8, 17]. The threshold of ≥1 g/d historically proposed to identify high-risk patients with IgAN [8, 9, 11], is still typically used as an inclusion criterion in randomized controlled trials [17] and currently considered as a ‘reasonable treatment target’ by the 2021-KDIGO guidelines [18].

Patients with IgAN and proteinuria <1 g/d are considered to have a more favorable prognosis [19], but their long-term health trajectories have been insufficiently studied [9, 10, 16]. Besides being a marker of more aggressive disease, proteinuria per se is directly nephrotoxic and further contributes to irreversible kidney damage [20, 21]. Clinical evidence and observational studies report that patients with IgAN and low-grade proteinuria may also progress [16, 22–24], supporting the hypothesis that a lower proteinuria target might be more clinically relevant [10, 15, 16]. However, available studies have generally been limited by small sample sizes [9, 19, 22, 23]. Other studies were conducted in young patients with normal kidney function or early stages of chronic kidney disease (CKD) [9, 10, 16], or with generally high levels of proteinuria [9, 16] and, thus, not enough powered to evaluate risks below 1 g/d [9]. Another limitation of previous studies is the evaluation of either proteinuria or the semi-quantitative urine dipstick [12], both of which are less precise than directly measured albuminuria [25, 26].

Identifying the optimal target of proteinuria in patients with IgAN is of importance for risk stratification, as well as tailoring monitoring and treatment strategies. In this study, using a nationwide contemporary cohort of patients with IgAN referred to nephrologist care, we evaluated the risk of kidney outcomes across the spectrum of urine albumin-to-creatinine ratio (uACR).

## MATERIALS AND METHODS

### Data source

The Swedish Renal Registry (SRR-CKD) is a nationwide registry of patients attending nephrology specialist care in Sweden. It collects a large set of clinical and biological data of CKD patients with an incident estimated glomerular filtration rate (eGFR) <30 mL/min per 1.73 m², but also encourages the enrollment of patients in earlier stages of CKD. Nearly all nephrology clinics in Sweden (98%) report to the SRR and it has been estimated that 75%–96% of all eligible patients in CKD stages 4–5 are enrolled [27]. SRR collects information on clinical and biological data, including eGFR, uACR, and etiology of kidney disease classified according to the European Renal Association Primary Renal Disease (ERA-PRD) coding system [28]. Information on whether or not the diagnosis was based on a kidney biopsy was available after 2012, when the ERA updated the coding classification. Approximately 90% of patients in SRR receive a specific kidney disease diagnosis.

Through each citizen's unique personal identification number, the SRR-CKD was linked to other government-run registries including: the National Patient Registry, which has provided information on all out-patient specialist consultations and hospitalizations occurring in Swedish healthcare since 1997; the Prescribed Drug Registry [29], which records information on prescribed drugs dispensed at any Swedish pharmacy; and the Death Registry [30], which provides information on date and causes of death. Government-run registries are considered to have no or minimal loss to follow-up. The study was approved by the Regional Ethical Review Board in Stockholm and the Swedish National Board of Health and Welfare (project number 2018/1591-31/2 and 2022-04 594).

### Study population

The study population consisted of adult patients with non-dialysis CKD due to primary IgAN registered in SRR-CKD between 1 January 2005 and 31 December 2021. Patients with secondary forms of IgAN (Figure S1), isolated hematuria and/or proteinuria without confirmation of IgAN, and patients with history of transplantation were excluded. The study design is presented in Figure S1.

We used two distinct cohorts. The first cohort included all patients with primary IgAN and at least one uACR measurement. The first recorded visit in the registry constituted the index date and the beginning of the follow-up. For the second cohort, the study population was restricted to patients with a second recorded visit within 12 ± 6 months that included an uACR measurement. In this second cohort, the index date was defined as the date of the second visit.

### Exposure

The study exposure was uACR, measured in an out-patient setting as a part of routine monitoring check-up. Urine albumin and creatinine concentrations were measured on either a first morning void or a random spot urine sample. uACR was calculated as urine albumin concentration divided by urine creatinine concentration and expressed in g/g (conversion factor: 1 mg/mmol = 8.84 mg/g = 0.00884 g/g, Table S1). Although uACR measurements were performed at different laboratories across Sweden, all the laboratory departments are audited annually by the Swedish Government-run agency EQUALIS to ensure reproducibility and national standardization across healthcare systems.

In the first cohort, we evaluated uACR at baseline (i.e. the first recorded uACR measurement in SRR), both as a continuous variable and as categories (<0.3, 0.3–0.5, 0.5–1.0, 1.0–1.5, 1.5–2.0, and ≥2.0 g/g). uACR lower than 0.3 g/g (i.e. KDIGO A1-A2), usually defined as clinical remission in other primary glomerular diseases [18], was considered the reference category.

In the second cohort, the exposure was the 1-year change in uACR, calculated as the ratio of the second over the first uACR measurement. The 1-year change in uACR was evaluated as a continuous variable (expressed on the log base 2 scale: the increase in one log_2_ unit corresponding to doubling uACR) and as categories (≥2-fold decrease, stable [reference category] and ≥2-fold increase).

### Covariates

Study covariates were derived at index date in each of the cohorts and included demographics, comorbidities, clinical and biological parameters, ongoing medications, and recent healthcare use (Table S2, Figure S1). Comorbidities included hypertension, diabetes mellitus, history of myocardial infarction, cerebrovascular disease, peripheral vascular disease, heart failure, arrhythmia, and history of acute kidney injury. Clinical parameters included systolic and diastolic blood pressure, body mass index, and laboratory measurements (hemoglobin, C-reactive protein, serum albumin, and eGFR using the CKD-EPI_2009_ equation). Ongoing medications, meaning the dispensed drugs from any pharmacy within the 6 months before index date, included renin-angiotensin system inhibitors (RASi), beta-blockers, calcium channel blockers, diuretics, corticosteroids, oral immunosuppressive drugs, antiplatelet therapy, and lipid-lowering drugs. Healthcare use considered the number of all-cause and CV-related hospitalizations and the number of out-patient visits in the year before the index date.

### Outcomes

The primary study outcome was major adverse kidney events (MAKE), a composite of kidney replacement therapy (KRT), and experiencing a decline in eGFR > 30% from baseline. KRT was defined as the start of maintenance dialysis or pre-emptive transplantation. The secondary outcomes included the single components of MAKE. Patients were followed until a kidney event, death, or the end of follow-up (31 December 2021).

### Statistical analyses

Baseline characteristics are presented overall and stratified by uACR categories. Categorical variables are presented as frequency and percentage, and continuous variables are presented as mean (standard deviation) or median (Q1–Q3, first–third quartile), as appropriate. Baseline conditions associated with uACR were analyzed using a linear regression model. Using all subsequent eGFR and uACR measurements registered during follow-up, we estimated the slopes of eGFR and uACR using linear mixed-effect regression models with random intercept and slope, and represented them graphically by baseline uACR category.

Adjusted cumulative incidence curves were estimated using the Aalen–Johansen approach, considering death as a competing risk. The relationships between uACR (treated as a continuous variable) and adverse kidney outcomes were assessed using restricted cubic splines and graphically depicted. Hazard ratios (HR) associated with uACR were estimated using Cox proportional hazards regression models. The models were adjusted on the following confounders, selected *a priori* on the basis of current knowledge and biological plausibility: age, sex, hypertension, diabetes mellitus, history of myocardial infarction, cerebrovascular disease, peripheral artery disease, heart failure, arrhythmia, acute kidney injury, systolic and diastolic blood pressure, baseline eGFR, hemoglobin, CRP, serum albumin, phosphate, ongoing use of RASi, mineralocorticoid receptor antagonists, statins, antiplatelet therapy, corticosteroids, immunosuppressive therapy, all-cause hospitalizations, and number of out-patient visits. Models evaluating the relationship between 1-year change in uACR and adverse kidney outcomes were additionally adjusted for baseline uACR. The proportional hazards assumption was checked using log(–log[S]) and Schoenfeld residuals against time.

As a sensitivity analysis, we re-ran the models restricting our study population to patients with biopsy-proven IgAN (*N* = 1209). We also explored the impact of microalbuminuria on kidney outcomes, splitting the reference category into <0.03 g/g (KDIGO A1, reference) and 0.03–0.3 g/g (A2) and keeping the other categories as explained previously.

Study covariates had no missing values except for blood pressure, hemoglobin, serum albumin, and phosphate, which were missing in <11%, and for CRP, which was missing in 26%. Because these clinical assessments are part of the monitoring in routine clinical practice in CKD, we assumed they were missing at random due to a lack of reporting into the registry. Multiple imputations were performed for missing covariates using the chained equation algorithm (50 imputed datasets, 20 iterations). Results from the 50 Cox models were pooled according to Rubin's rule.

The STROBE statement was followed for reporting observational studies [31]. All statistical analyses were conducted using R v.3.6.3 software.

## RESULTS

### Clinical characteristics of patients by uACR categories

**Figure 1:**
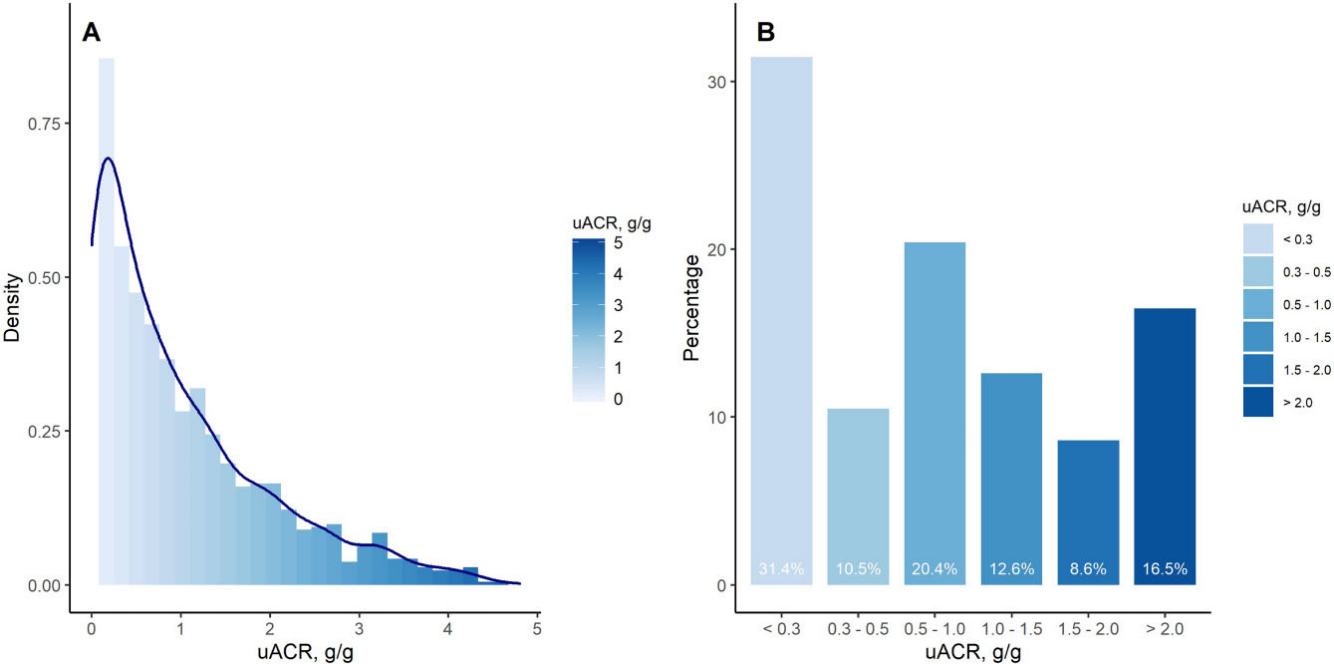
Distribution of baseline uACR (**A**) and percentage of patients by category of uACR (**B**).

We identified 1269 patients with primary IgAN, which was based on kidney biopsy in 95.3%. Their median age was 53 [Q1–Q3: 41; 66] years, 74% were men, and mean eGFR was 33±20 ml/min/1.73 m². As many as 38% of patients had uACR >1 g/g. An additional 10.5% of patients had uACR 0.3–0.5 g/g, and 20.4% had uACR 0.5–1.0 g/g (Fig. 1). Across higher categories of uACR, patients were younger, more often men, had a higher prevalence of hypertension, diabetes and acute kidney injury, had lower eGFR and serum albumin level, and were prescribed RASi less often (Table 1). The baseline conditions associated with higher uACR levels included younger age, male sex, diabetes comorbidity, higher systolic blood pressure, lower eGFR and serum albumin, and absence of RASi use (Table S3).

**Table 1:** Baseline characteristics of patients with IgA nephropathy (*N* = 1269) enrolled in the SRR-CKD.

		Categories of uACR					
	Overall	<0.3 g/g	[0.3–0.5[ g/g	[0.5–1.0[ g/g	[1.0–1.5[ g/g	[1.5–2.0[ g/g	≥2.0 g/g
	*N* = 1269 (100%)	*N* = 399 (31.4%)	*N* = 133 (10.5%)	*N* = 259 (20.4%)	*N* = 160 (12.6%)	*N* = 109 (8.6%)	*N* = 209 (16.5%)
Demographics and clinical data							
Women	329 (25.9)	112 (28.1)	45 (33.8)	64 (24.7)	38 (23.8)	18 (16.5)	52 (24.9)
Age, years [Q1–Q3]	53 [41, 66]	60 [47, 68]	52 [41, 66]	53 [39, 64]	51 [39, 62]	48 [35, 63]	49 [36, 61]
Body mass index, kg/m²	28.5 (5.7)	28.6 (5.6)	27.2 (4.9)	28.3 (5.8)	28.9 (5.4)	29.2 (7.0)	28.7 (5.8)
Systolic blood pressure, mmHg	136 (18.4)	129 (15.3)	132 (17.6)	134 (16.2)	137 (17.7)	141 (18.9)	148 (19.5)
Diastolic blood pressure, mmHg	82 (11.1)	78 (9.5)	80 (9.6)	81 (10.7)	84 (10.1)	83 (11.8)	88 (12.5)
Comorbidies							
Hypertension	1044 (82.3)	303 (75.9)	107 (80.5)	216 (83.4)	137 (85.6)	95 (87.2)	186 (89.0)
Diabetes mellitus	171 (13.5)	51 (12.8)	17 (12.8)	31 (12.0)	17 (10.6)	18 (16.5)	37 (17.7)
Any Cardiovascular disease	137 (10.8)	45 (11.3)	15 (11.3)	27 (10.4)	15 (9.4)	12 (11.0)	23 (11.0)
Myocardial infarction	55 (4.3)	22 (5.5)	6 (4.5)	13 (5.0)	5 (3.1)	1 (0.9)	8 (3.8)
Peripheral vascular disease	19 (1.5)	6 (1.5)	3 (2.3)	5 (1.9)	1 (0.6)	0 (0.0)	4 (1.9)
Cerebrovascular disease	87 (6.9)	30 (7.5)	9 (6.8)	14 (5.4)	10 (6.2)	11 (10.1)	13 (6.2)
Heart failure	72 (5.7)	28 (7.0)	4 (3.0)	12 (4.6)	12 (7.5)	5 (4.6)	11 (5.3)
Arrhythmia	86 (6.8)	40 (10.0)	7 (5.3)	15 (5.8)	11 (6.9)	2 (1.8)	11 (5.3)
Acute kidney injury	167 (13.2)	37 (9.3)	19 (14.3)	31 (12.0)	23 (14.4)	16 (14.7)	41 (19.6)
Biological values							
Hemoglobin, g/dl	12.7 (1.8)	13.2 (1.7)	12.6 (1.6)	12.7 (1.8)	12.6 (1.8)	12.5 (1.9)	12.0 (1.5)
C-reactive protein, mg/l	5.8 (10.1)	5.8 (8.9)	7.2 (12.0)	5.7 (13.4)	5.5 (10.0)	5.4 (8.1)	5.4 (7.4)
Serum Albumin, g/l	36 (4.6)	38 (4.1)	37 (3.5)	37 (3.9)	36 (3.8)	34 (4.0)	32 (4.6)
eGFR, ml/min per 1.73 m²	33 (19.8)	41 (23.6)	33 (17.5)	32 (17.2)	30 (17.5)	27 (15.1)	25 (13.9)
eGFR category							
>60 ml/min per 1.73 m²	107 (8.4)	65 (16.3)	10 (7.5)	14 (5.4)	10 (6.2)	3 (2.8)	5 (2.4)
45–59 ml/min per 1.73 m²	130 (10.2)	61 (15.3)	15 (11.3)	30 (11.6)	8 (5.0)	8 (7.3)	8 (3.8)
30–44 ml/min per 1.73 m²	312 (24.6)	111 (27.8)	36 (27.1)	65 (25.1)	39 (24.4)	25 (22.9)	36 (17.2)
15–29 ml/min per 1.73 m²	575 (45.3)	146 (36.6)	62 (46.6)	123 (47.5)	76 (47.5)	50 (45.9)	118 (56.5)
<15 ml/min per 1.73 m²	145 (11.4)	16 (4.0)	10 (7.5)	27 (10.4)	27 (16.9)	23 (21.1)	42 (20.1)
uACR, g/g [Q1–Q3]	0.7 [0.2, 1.5]	0.1 [0.0, 0.2]	0.4 [0.3, 0.4]	0.7 [0.6, 0.9]	1.2 [1.1, 1.3]	1.7 [1.6, 1.9]	2.8 [2.3, 3.4]
Ongoing medications							
RASi	1055 (83.1)	332 (83.2)	116 (87.2)	221 (85.3)	134 (83.8)	91 (83.5)	161 (77.0)
Calcium channel blockers	685 (54.0)	168 (42.1)	67 (50.4)	151 (58.3)	88 (55.0)	73 (67.0)	138 (66.0)
Diuretics	554 (43.7)	171 (42.9)	54 (40.6)	109 (42.1)	64 (40.0)	46 (42.2)	110 (52.6)
Beta-blockers	551 (43.4)	166 (41.6)	51 (38.3)	109 (42.1)	66 (41.2)	55 (50.5)	104 (49.8)
Corticosteroids	243 (19.1)	74 (18.5)	21 (15.8)	49 (18.9)	29 (18.1)	21 (19.3)	49 (23.4)
Oral immunosuppressive therapy	53 (4.2)	16 (4.0)	8 (6.0)	8 (3.1)	5 (3.1)	4 (3.7)	12 (5.7)
Antiplatelets	149 (11.7)	42 (10.5)	19 (14.3)	31 (12.0)	15 (9.4)	14 (12.8)	28 (13.4)
Lipid-lowering therapy	574 (45.2)	185 (46.4)	64 (48.1)	110 (42.5)	73 (45.6)	47 (43.1)	95 (45.5)
Healthcare use in the previous year							
No. of hospitalizations, median [Q1–Q3]	0 [0, 1]	0 [0, 1]	0 [0, 1]	0 [0, 1]	0 [0, 1]	0 [0, 1]	1 [0, 1]
≥1 hospitalization, *n* (%)	467 (36.8)	110 (27.6)	47 (35.3)	92 (35.5)	60 (37.5)	53 (48.6)	105 (50.2)
≥1 CV-related hospitalization, *n* (%)	38 (3.0)	13 (3.3)	2 (1.5)	13 (5.0)	5 (3.1)	1 (0.9)	4 (1.9)
No. of out-patient visits [Q1–Q3]	2 [1, 4]	2 [1, 4]	2 [1, 4]	2 [1, 4]	3 [1, 5]	2 [1, 5]	3 [1, 4]

Categorical variables are reported as frequencies (and percentages), and continuous variables are reported as mean (SD) or median [Q1–Q3: first and third quartile].

### Evolution of eGFR and uACR over the study period

**Figure 2:**
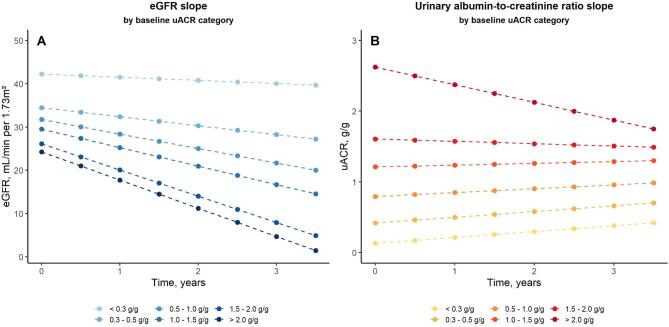
Evolution of eGFR and uACR in patients with IgA nephropathy (*N* = 1269), according to baseline categories of uACR. The evolution eGFR and uACR was estimated using linear mixed models with random intercept and slope, accounting for all eGFR and uACR measurements, respectively, over the study period.

During a median follow-up of 5.5 [Q1-Q3: 2.8; 9.2] years, 8490 and 6848 subsequent measurements of eGFR and uACR were, respectively, recorded. The mean annual eGFR decline was overall of –3.06 [95%CI: −3.36; −2.77] ml/min/1.73 m² per year, ranging from −0.74 [−1.18; −0.29] ml/min/1.73 m² per year in patients with uACR < 0.3 g/g to −6.52 [−7.26; −5.78] ml/min/1.73 m² per year in those with uACR ≥ 2.0 g/g. While patients with baseline uACR ≥ 2.0 g/g had an annual uACR decline of −0.25 [–0.30; −0.20] g/g per year, uACR remained stable in patients with uACR 1.0–2.0 g/g and showed an increasing trend in patients with baseline uACR < 1.5 g/g (Fig. 2, Table S4).

### uACR and risk of adverse kidney events

**Figure 3:**
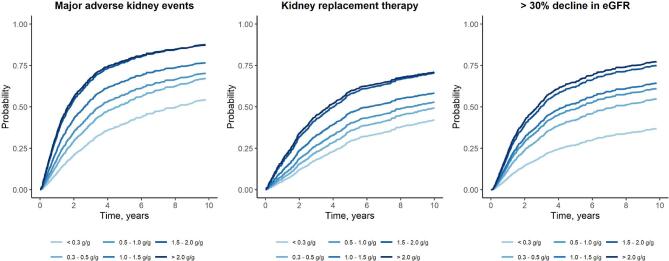
Adjusted cumulative incidence curves for MAKE and its individual components, according to categories of baseline uACR (*N* = 1269). The cumulative incidence curves were estimated using the Aalen–Johansen estimator considering the competing risk of death and were adjusted for age, sex, hypertension, diabetes, history of myocardial infarction, cerebrovascular disease, peripheral artery disease, heart failure, arrhythmia, acute kidney injury, systolic and diastolic blood pressure, eGFR, hemoglobin, CRP, serum albumin, phosphate, RASi, mineralocorticoid receptor antagonists, statins, antiplatelet therapy, corticosteroids, immunosuppressive therapy, all-cause hospitalizations, and number of out-patient visits in the year prior.

During follow-up, 667 MAKE and 517 KRT events occurred, and 528 patients experienced an eGFR decline of a magnitude >30% from baseline. Adjusted absolute risks of kidney events were higher across more severe uACR categories (Table 2, Fig. 3). For example, the 5-year risk of MAKE was 39.6% in patients with uACR < 0.3 g/g and 77.6% in patients with uACR ≥ 2.0 g/g.

**Table 2:** Absolute risks and HR for the rates of MAKE and its individual components according to baseline uACR (*N* = 1269).

	Number of events, *n*/*N*	Adjusted 5-year absolute risk (95% CI)*	Crude HR (95% CI)	Adjusted HR* (95% CI)
MAKE				
For each 0.1 g/g higher in uACR	667/1269		1.04 (1.04–1.05)	1.03 (1.02–1.04)
Categories of uACR				
<0.3 g/g	117/399	39.6 (34.6; 44.6)	Ref.	Ref.
[0.3–0.5[ g/g	63/133	51.6 (45.6; 57.7)	1.88 (1.39–2.56)	1.56 (1.14–2.14)
[0.5–1.0[ g/g	144/259	57.1 (52.1; 62.1)	2.75 (2.16–3.52)	2.04 (1.58–2.64)
[1.0–1.5[ g/g	95/160	65.3 (59.7; 70.9)	3.82 (2.90–5.02)	2.82 (2.10–3.78)
[1.5–2.0[ g/g	81/109	76.7 (70.1; 83.3)	6.57 (4.93–8.77)	4.21 (3.08–5.74)
≥2.0 g/g	167/209	77.6 (71.8; 83.4)	7.48 (5.88–9.51)	4.53 (3.36–6.11)
KRT				
For each 0.1 g/g higher in uACR	517/1269		1.04 (1.04–1.05)	1.03 (1.02–1.04)
Categories of uACR				
< 0.3 g/g	78/399	29.2 (24.6; 33.9)	Ref.	Ref.
[0.3–0.5[ g/g	43/133	35.5 (30.2; 40.7)	1.71 (1.18–2.48)	1.39 (0.94–2.03)
[0.5–1.0[ g/g	103/259	40.0 (35.5; 44.6)	2.62 (1.95–3.52)	1.84 (1.35–2.51)
[1.0–1.5[ g/g	76/160	46.5 (40.9; 52.1)	3.91 (2.84–5.37)	2.59 (1.84–3.64)
[1.5–2.0[ g/g	70/109	57.7 (50.3; 65.2)	6.59 (4.76–9.13)	4.14 (2.89–5.91)
≥2.0 g/g	147/209	59.5 (53.6; 65.3)	7.78 (5.89–10.27)	4.65 (3.28–6.59)
>30% decline in eGFR				
For each 0.1 g/g higher in uACR	528/1269		1.03 (1.02–1.03)	1.02 (1.01–1.03)
Categories of uACR				
<0.3 g/g	97/399	26.8 (22.1; 31.4)	Ref.	Ref.
[0.3–0.5[ g/g	56/133	41.8 (34.3; 49.3)	1.96 (1.41–2.72)	1.76 (1.26–2.47)
[0.5–1.0[ g/g	120/259	48.4 (42.5; 54.3)	2.48 (1.90–3.25)	2.20 (1.66–2.91)
[1.0–1.5[ g/g	74/160	51.8 (44.1; 59.5)	2.81 (2.07–3.80)	2.45 (1.78–3.39)
[1.5–2.0[ g/g	60/109	61.8 (52.0; 71.6)	3.90 (2.83–5.39)	3.20 (2.25–4.56)
≥2.0 g/g	121/209	64.7 (55.9; 73.5)	4.06 (3.11–5.31)	3.47 (2.47–4.87)

Cox models were adjusted for age, sex, hypertension, diabetes, history of myocardial infarction, cerebrovascular disease, peripheral artery disease, heart failure, arrhythmia, acute kidney injury, systolic and diastolic blood pressure, eGFR, hemoglobin, CRP, serum albumin, phosphate, RASi, mineralocorticoid receptor antagonists, statins, antiplatelet therapy, corticosteroids, immunosuppressive therapy, all-cause hospitalizations, and number of out-patient visits.

*5-year absolute risks show the percentage of patients experiencing kidney event by uACR category, considering the competing risk of death.

When uACR was expressed as a continuous variable, every 0.1 g/g higher in uACR was associated with a 3% higher risk of MAKE [adjusted HR 1.03 (95%CI 1.02;1.04)]. In categories, and compared with uACR < 0.3 g/g, any higher uACR category was strongly and incrementally associated with the risk of MAKE, with HR ranging from 1.56 [1.14; 2.14] in patients with uACR 0.3–0.5 g/g to 4.53 [3.36; 6.11] in patients with uACR ≥ 2.0 g/g. Similar graded relationships were observed for KRT [HR ranging from 1.39 (0.94; 2.03) to 4.65 (3.28; 6:59)], and for >30% decline in eGFR [HR ranging from 1.76 (1.26; 2.47) to 3.47 (2.47; 4.87)] (Table 2). The ‘dose–effect’ relationship was also observed in the low-ranges of uACR (Table S5). Restricted cubic splines depicted a linear relationship between uACR and the risk of all study outcomes (Table 2, Figure S2). Similar results were observed when the study population was restricted to patients with biopsy-proven IgAN (Table S6).

### Clinical characteristics of patients by 1-year change in uACR

From the initial cohort of patients with IgAN, we identified 785 patients [median age 56 (Q1–Q3: 44; 68) years, 74.4% men, eGFR 31±19 ml/min/1.73 m²] with repeated uACR measurements. Of these, 450 (57.3%) patients had a stable uACR, 198 (25.2%) experienced a decrease in uACR of 2-fold or greater, and 137 (17.5%) experienced an increase in uACR of 2-fold or greater within a year. Compared with patients exhibiting a stable uACR, those with 2-fold decrease were younger, more often women, had a higher baseline eGFR and a lower uACR level, and were more often prescribed corticosteroids and immunosuppressive therapy. By contrast, patients with a 2-fold increase in uACR had higher CRP and uACR levels despite similar RASi use and were receiving corticosteroids and immunosuppressive therapy less often (Table S7).

### 1-year change in uACR and adverse kidney events

Over median follow-up of 4.9 [Q1–Q3: 2.8; 8.5] years, 448 MAKE and 318 KRT events occurred, and 406 patients experienced an eGFR decline of a magnitude >30% from baseline. Compared with patients with stable uACR, the risk of kidney events was lower when uACR decreased by 2-fold (adjusted HR ranging from 0.47 to 0.49), and higher when uACR increased by 2-fold (HR from 1.18 to 2.56), irrespective of baseline uACR (Table 3). Similar results were observed when a change in uACR was expressed as a continuous variable (Table 3).

**Table 3:** HR for the rates of MAKE and its individual components according to 1-year change in uACR (*N* = 785).

	Number of events *n*/*N*	Crude HR (95%CI)	Adjusted HR* (95%CI)
MAKE			
Per fold increase in uACR (log_2_)	448/785	1.10 (1.04–1.15)	1.27 (1.20–1.35)
Fold change in uACR, categories			
2-fold decrease in uACR	91/198	0.67 (0.53–0.85)	0.47 (0.36–0.62)
Stable uACR	275/450	Ref.	Ref.
2-fold increase in uACR	82/137	0.93 (0.73–1.20)	1.75 (1.31–2.33)
KRT			
Per fold increase in uACR (log_2_)	318/785	1.12 (1.05–1.19)	1.48 (1.36–1.62)
Fold change in uACR, categories			
2-fold decrease in uACR	63/198	0.71 (0.53–0.94)	0.49 (0.36–0.68)
Stable uACR	194/450	Ref.	Ref.
2-fold increase in uACR	61/137	1.06 (0.79–1.41)	2.56 (1.79–3.65)
>30% decline in eGFR			
Per fold increase in uACR (log_2_)	406/785	1.10 (1.05–1.16)	1.19 (1.11–1.27)
Fold change in uACR, categories			
2-fold decrease in uACR	76/198	0.57 (0.45–0.74)	0.49 (0.37–0.65)
Stable uACR	261/450	Ref.	Ref.
2-fold increase in uACR	69/137	0.78 (0.60–1.02)	1.18 (0.87–1.60)

1-year change in uACR was analyzed both as a continuous variable (and expressed on the log base 2 scale—the increase in one log_2_ unit corresponds to doubling uACR), and as categories (≥2-fold decrease, stable, ≥2-fold increase). Cox models were adjusted for age, sex, hypertension, diabetes, history of myocardial infarction, cerebrovascular disease, peripheral artery disease, heart failure, arrhythmia, acute kidney injury, systolic and diastolic blood pressure, eGFR, baseline albumin-to-creatinine ratio, hemoglobin, CRP, serum albumin, phosphate, RASi, mineralocorticoid receptor antagonists, statins, antiplatelet therapy, corticosteroids, immunosuppressive therapy, all-cause hospitalizations, and number of out-patient visits.

## DISCUSSION

In this large cohort of patients with IgAN-related advanced CKD, we show that uACR level was strongly and incrementally associated with the risk of adverse kidney outcomes. Compared to uACR < 0.3 g/g, we observed substantial risk of adverse kidney outcomes among patients with uACR between 0.3 and 1.0 g/g: a population currently considered at low risk of CKD progression. Any reduction in uACR within one year was also independently associated with better kidney outcomes, irrespective of baseline uACR. Our findings were robust across kidney outcome definitions and when considering uACR as a continuous variable. Collectively, these findings provide a rationale to re-evaluate the current risk-based treatment target (i.e. proteinuria <1 g/d) in these patients.

Our findings expand previous work conducted in patients with normal kidney function or early stage of CKD, and high levels of proteinuria [9, 10, 12, 15, 16, 32]. Novelties in our analysis include the evaluation of patients with IgAN-related advanced CKD, and the use of the full range of uACR with narrower uACR categories. We observed an independent and rather linear direct relationship between uACR and kidney outcomes, with no effect modification above versus below 1 g/g. In our study, the 5-year absolute risks of KRT were high, both for patients with high and low-grade uACR, ranging from 35.5% in patients with uACR 0.3–0.5 g/g to 60% in patients with ≥2.0 g/g. This may reflect in part their advanced CKD at enrollment. However, our findings align with a recent pivotal study from the UK National Registry of Rare Diseases (RaDaR) cohort that enrolled 2299 patients with IgAN and less severe CKD (mean eGFR 55 ml/min/1.73 m²), and reported that 30% of patients with a time-averaged proteinuria of 0.44 to 0.88 g/g and 20% of those with time-averaged proteinuria below 0.44 g/g would reach kidney failure within 10 years [16].

In terms of relative risks, our results show that compared to uACR < 0.3 g/g, any higher uACR level was independently associated with a higher rate of adverse kidney events. In patients with low-grade uACR, we observed a 39% and 84% increased risk of KRT in patients with uACR 0.3–0.5 g/g and 0.5–1.0 g/g, respectively, compared to those whose uACR was <0.3 g/g. Reich *et al.* failed to observe a difference in kidney outcome for proteinuria below 1 g/d [9]. However, their sample size (*N* = 542) was low and may have biased their observations. Subsequent studies have challenged this [10, 12, 15, 16]. For example, a Chinese study conducted in 1155 patients with IgAN and normal kidney function observed that, although the cut-off for predicting an unfavorable kidney outcome was a time-averaged proteinuria of 0.97 g/d, proteinuria level of 0.5 g/d was associated with better kidney outcome compared to both 0.5–1.0 and 1.0 g/d [10]. Our study thus supports and expands these previous works to North-European patients with advanced CKD.

In our study, we also observed that any reduction in uACR within one year, regardless of baseline uACR levels, was associated with improved kidney outcomes. As in all observational studies, causality cannot be inferred since the reasons behind albuminuria improvement or worsening are not known. However, there is a lot of evidence to support causality because of biological plausibility [20, 21], as well as data from clinical trials [8, 17] and epidemiological studies [8–15, 16]. Regardless of the underlying etiology of nephropathy, proteinuria *per se* is nephrotoxic, mainly by promoting inflammation in tubular epithelial cells, leading to interstitial fibrosis and acceleration of kidney disease progression [20, 21]. The importance of proteinuria reduction in delaying CKD progression has been demonstrated in other glomerular diseases, such as membranous nephropathy [33] and focal segmental glomerulosclerosis (FSGS, [34]) where ‘complete remission’ is defined as proteinuria <0.3 g/d [18]. However, what constitutes a complete remission—i.e. a treatment response associated with a low rate of persistent or recurrent disease activity and excellent long-term kidney and patient survival—in the setting of IgAN is not well defined, and may include criteria other than proteinuria alone. Likewise, the optimal uACR level and duration that confers a protective effect in IgAN remains to be determined. Albuminuria is associated with CKD progression, but does not necessarily mean active inflammatory IgAN (i.e. with a higher probability to respond to immunosuppressive therapy). Thus, differentiating patients with active IgAN from those with chronic kidney damage is critical for adequate individual risk stratification and avoiding unnecessary exposure to immunosuppressive medication [18], and more specific markers reflecting the activity and chronicity of the disease are eagerly awaited.

Additional strengths of our study include: a national representative cohort of well-phenotyped patients with IgAN attending nephrologist care; a long-term follow-up with virtually no loss to follow-up thanks to data linkage with high-quality registries; and the setting of the Swedish universal tax-funded healthcare that minimizes selection bias from disparate access to healthcare. Our study also has limitations: uACR was measured on a spot urine sample, which although better than urinary protein excretion, and well correlated with 24-h urinary albumin excretion [25, 26], is not the gold standard to assess uACR and may be affected by interlaboratory and interindividual variability. However, uACR measured on a spot urine sample does not show a lower performance than urinary albumin excretion [35] and is commonly used in routine practice for clinical decision-making. Further, patients were included at the time of their registration in SRR-CKD—not necessarily at the time of diagnosis—and we lacked information on hematuria, histological features, and MEST-C score [36]. Our study population consisted in patients with an eGFR ∼30 ml/min/1.73 m², and thus, with probably chronic and irreversible kidney damage. We cannot rule out the possibility of misclassification bias in the 4.7% of patients whose IgAN was not confirmed by a kidney biopsy, and in patients enrolled before 2012, when ERA changed its coding system. Finally, our study was conducted in patients of predominantly Caucasian ethnicity, and with a more advanced stage of CKD and a higher prevalence of comorbidities than those recruited in pivotal trials. Thus, extrapolation of our findings to other populations should be done with caution.

Our study has clinical implications. Because changes in uACR predicted subsequent kidney risks beyond current uACR levels, our data support long-term monitoring of uACR to inform decisions for risk stratification and treatment. The 2021-KDIGOs guidelines recommend RASi to be prescribed to patients with IgAN and proteinuria >0.5 g/d irrespective of whether they have hypertension [18]. In our study, we observed that the overall rate of patients treated with RASi (83.1%) was rather low. Interestingly, the highest rates (83.2% and 87.2%) were seen in those with the weakest indication (uACR < 0.5 g/g), whereas 23% of patients with uACR ≥2 g/g (who also have a lower eGFR level) were not receiving RASi. This is likely explained by the fact that clinicians are often reluctant to prescribe RASi in advanced CKD, and thus identifying potentially a clinical gap that needs correction. Finally, the threshold of proteinuria ≥1 g/d, initially proposed to identify patients with IgAN at high risk for CKD progression [8, 9, 11], has become a classic inclusion criterion in pivotal clinical trials, [17] and a now recommended ‘reasonable treatment target’ by guidelines [18]. Our findings support rising claims [10, 15, 16] on the need of re-considering the current proteinuria treatment target.

To conclude, this nationwide study of patients with IgAN-related advanced CKD shows that uACR is independently associated with the risk of adverse kidney outcomes, also in patients with low-grade uACR, a population traditionally considered at low risk of CKD progression. Any reduction in uACR, irrespective of baseline uACR, is associated with better kidney outcomes.

## Supplementary Material

gfae085_Supplemental_File

## Data Availability

The data underlying this article cannot be shared publicly due to the privacy of individuals that participated in the study. The data may be shared on reasonable request for academic research collaborations that fulfill GDPR as well as national and institutional ethics regulations and standards by contacting Dr Marie Evans (marie.evans@ki.se).
