# Supplementary material for: Albuminuria predicts kidney events in IgA nephropathy
Source: Nephrol Dial Transplant. 2024 Apr 30;40(3):465–74. doi: 10.1093/ndt/gfae085 (PMC11997806; doi:10.1093/ndt/gfae085)
Supplement: gfae085_Supplemental_File [file gfae085_Supplemental_File.docx]

**SUPPLEMENTARY MATERIAL**

**Albuminuria and long-term kidney outcomes in patients with IgA Nephropathy:**

**a nationwide study**

Anne-Laure Faucon, Sigrid Lundberg, Stefania Lando, Julia Wijkström, Mårten Segelmark, Marie Evans, Juan-Jesús Carrero.

TABLE OF CONTENTS

[Table S1. Conversion table 2](#_Toc159789450)

[Table S2. Definitions of comorbidities and medications. 3](#_Toc159789451)

[Table S3. Baseline conditions associated with urine albumin-to-creatinine in patients with IgA nephropathy 4](#_Toc159789452)

[Table S4. Annual crude eGFR and uACR slopes over the study period, by baseline albumin-to-creatinine category, in patients with IgA nephropathy (N=1269). 5](#_Toc159789453)

[Table S5. Adjusted hazard ratios for the rates of kidney outcomes according to baseline urine albumin-to-creatinine ratio, exploring low-ranges of urine albumin-to-creatinine ratio. 6](#_Toc159789454)

[Table S6. Adjusted hazard ratios for the rates of kidney outcomes according to baseline urine albumin-to-creatinine ratio, in patients with biopsy proven IgAN (N=1209). 7](#_Toc159789455)

[Table S7. Characteristics of patients with IgA nephropathy by category of 1-year change in urine albumin-to-creatinine ratio (uACR) (N=785) 8](#_Toc159789456)

[Figure S1. Study design. 9](#_Toc159789457)

[Figure S2. Restricted cubic splines depicting the rates of major adverse kidney events and its individual components across the continuum of baseline urine albumin-to-creatinine ratio (N=1269). 10](#_Toc159789458)

# Table S1. Conversion table

| **uACR, mg/mmol** | **uACR, mg/g** | **uACR, g/g** |
| --- | --- | --- |
| 33.9 | 300 | 0.3 |
| 56.5 | 500 | 0.5 |
| 113 | 1000 | 1.0 |
| 170 | 1500 | 1.5 |
| 226 | 2000 | 2.0 |

Conversion factor: 1 mg/mmol = 8.84 mg/g = 0.00884 g/g

# Table S2. Definitions of comorbidities and medications.

|  | **ICD-10 codes** | **ATC codes** |
| --- | --- | --- |
| **Comorbidities** |  |  |
| Diabetes mellitus | E10-E14 | A10 |
| Hypertension | I10-I15 | or antihypertensive drugs |
| Myocardial infarction | I21, I22, I25.2 |  |
| Heart failure | I11.0, I13.0, I13.2, I50 |  |
| Cerebrovascular disease | G45.9, I63, I64, I69 |  |
| Peripheral vascular disease | I70,I72, I73 |  |
| Arrythmia | I47-I49 |  |
| Acute kidney injury | N17 |  |
| **Medications** |  |  |
| Renin-angiotensin system inhibitors |  | C09A, C09C |
| Calcium-channel blockers |  | C08C, C08D |
| Beta-blockers |  | C07 |
| Diuretics |  | C03A, C03B, C03C, C03D |
| Corticosteroids |  | H02 |
| Oral immunosuppressive therapy |  | L04 |
| Antiplatelet therapy |  | B01AC |
| Lipid lowering therapy |  | C10 |

ICD-10: International Classification of Diseases tenth revision; ATC: Anatomic Therapeutic Chemical Classification System.

# Table S3. Baseline conditions associated with urine albumin-to-creatinine in patients with IgA nephropathy

|  | **Crude beta coefficients**  **(95% CI)** | **Adjusted beta coefficients**  **(95%CI)** |
| --- | --- | --- |
| Age, years | -0.17 (-0.23 ; -0.11)* | -0.25 (-0.31 ; -0.20)* |
| Sex, women | -0.09 (-0.23 ; 0.05) | -0.13 (-0.25 ; -0.01)* |
| Diabetes mellitus | 0.23 (0.05 ; 0.41)* | 0.24 (0.09 ; 0.40)* |
| Any cardiovascular disease | 0.03 (-0.17 ; 0.23) | 0.16 (-0.01 ; 0.33) |
| Acute kidney injury | 0.38 (0.20 ; 0.56)* | 0.08 (-0.07 ; 0.24) |
| Systolic BP, mmHg | 0.38 (0.33 ; 0.44)* | 0.24 (0.18 ; 0.31)* |
| Diastolic BP, mmHg | 0.31 (0.25 ; 0.37)* | 0.06 (-0.01 ; 0.13) |
| eGFR, mL/min/1.73m² | -0.29 (-0.35 ; -0.23)* | -0.20 (-0.25 ; -0.15)* |
| Serum albumin, g/L | -0.53 (-0.59 ; -0.48)* | -0.45 (-0.50 ; -0.40)* |
| RAS inhibitors | -0.23 (-0.40 ; -0.07)* | -0.20 (-0.33 ; -0.06)* |
| Corticosteroids | 0.22 (0.06 ; 0.38)* | -0.07 (-0.20 ; 0.07) |
| Oral immunosuppressive therapy | 0.19 (-0.12 ; 0.50) | -0.03 (-0.28 ; 0.23) |

Albumin-to-creatinine ratio is expressed in g/g. Beta coefficients were estimated using linear regression models. All continuous variables are modelled as per 1 standard deviation increase.

BP: blood pressure ; 95%CI: 95% confidence intervals ; eGFR: estimated glomerular filtration rate; RAS inhibitors: renin-angiotensin system inhibitors. * p-value < 0.05

# Table S4. Annual crude eGFR and uACR slopes over the study period, by baseline albumin-to-creatinine category, in patients with IgA nephropathy (N=1269).

|  | **eGFR slope, mL/min/1.73m² per year** | **uACR slope, g/g per year** |
| --- | --- | --- |
| **Total population** | -3.06 (-3.36 ; -2.77) | 0.02 (-0.14 ; 0.17) |
|  |  |  |
| **Baseline uACR categories** |  |  |
| < 0.3 g/g | -0.74 (-1.18 ; -0.29) | 0.08 (0.06 ; 0.11) |
| [0.3 - 0.5[ g/g | -2.07 (-2.82 ; -1.32) | 0.08 (0.04 ; 0.12) |
| [0.5 - 1.0[ g/g | -3.35 (-3.91 ; -2.79) | 0.05 (0.02 ; 0.09) |
| [1.0 - 1.5[ g/g | -4.28 (-5.04 ; -3.51) | 0.02 (-0.02 ; 0.07) |
| [1.5 - 2.0[ g/g | -6.07 (-7.07 ; -5.06) | -0.03 (-0.10 ; 0.03) |
| ≥ 2.0 g/g | -6.52 (-7.26 ; -5.78) | -0.25 (-0.30 ; -0.20) |

eGFR and uACR slope were estimated using a linear mixed model with random intercept and slope, accounting for all eGFR and uACR measurements over the study period. eGFR: estimated glomerular filtration rate; uACR: urine albumin-to-creatinine ratio.

# Table S5. Adjusted hazard ratios for the rates of kidney outcomes according to baseline urine albumin-to-creatinine ratio, exploring low-ranges of urine albumin-to-creatinine ratio.

|  | **n events / N** | **Adjusted HR***  **(95% CI)** |
| --- | --- | --- |
| **Major adverse kidney events** |  |  |
| Categories of uACR |  |  |
| < 0.03 g/g | 15 / 94 | Ref |
| [0.3 - 0.3[ g/g | 102 / 305 | 2.06 (1.19-3.58) |
| [0.3 - 0.5[ g/g | 63 / 133 | 2.85 (1.6-5.07) |
| [0.5 - 1.0[ g/g | 144 / 259 | 3.75 (2.17-6.49) |
| [1.0 - 1.5[ g/g | 95 / 160 | 5.18 (2.94-9.13) |
| [1.5 - 2.0[ g/g | 81 / 109 | 7.7 (4.33-13.68) |
| ≥ 2.0 g/g | 167 / 209 | 8.35 (4.71-14.79) |
| **Kidney replacement therapy** |  |  |
| Categories of uACR |  |  |
| < 0.03 g/g | 6 / 94 | Ref |
| [0.3 - 0.3[ g/g | 72 / 305 | 3.17 (1.36-7.37) |
| [0.3 - 0.5[ g/g | 43 / 133 | 3.79 (1.59-9.04) |
| [0.5 - 1.0[ g/g | 103 / 259 | 5.06 (2.18-11.76) |
| [1.0 - 1.5[ g/g | 76 / 160 | 7.15 (3.04-16.82) |
| [1.5 - 2.0[ g/g | 70 / 109 | 11.32 (4.79-26.77) |
| ≥ 2.0 g/g | 147 / 209 | 12.89 (5.45-30.5) |
| **>30% decline in eGFR** |  |  |
| Categories of uACR |  |  |
| < 0.03 g/g | 13 / 94 | Ref |
| [0.3 - 0.3[ g/g | 84 / 305 | 2.09 (1.16-3.78) |
| [0.3 - 0.5[ g/g | 56 / 133 | 3.23 (1.75-5.98) |
| [0.5 - 1.0[ g/g | 120 / 259 | 4.05 (2.25-7.28) |
| [1.0 - 1.5[ g/g | 74 / 160 | 4.53 (2.46-8.34) |
| [1.5 - 2.0[ g/g | 60 / 109 | 5.91 (3.16-11.05) |
| ≥ 2.0 g/g | 121 / 209 | 6.44 (3.46-11.97) |

Cox models were adjusted for age, sex, hypertension, diabetes, history of myocardial infarction, cerebrovascular disease, peripheral artery disease, heart failure, arrhythmia, acute kidney injury, systolic and diastolic blood pressure, eGFR, hemoglobin, CRP, serum albumin, phosphate, renin-angiotensin system inhibitors, mineralocorticoid receptor antagonists, statins, antiplatelet therapy, corticosteroids, immunosuppressive therapy, all-cause hospitalizations and number of out-patient visits.

MAKE: major adverse kidney events (a composite of kidney replacement therapy and >30% decline in eGFR).

# Table S6. Adjusted hazard ratios for the rates of kidney outcomes according to baseline urine albumin-to-creatinine ratio, in patients with biopsy proven IgAN (N=1209).

|  | **Adjusted HR* (95% CI)**  **- Main analysis -**  **N = 1269** | **Adjusted HR* (95% CI)**  **- Biopsy proven IgAN -**  **N = 1209** |
| --- | --- | --- |
| **Major adverse kidney events** |  |  |
| For each 0.1 g/g higher in uACR | 1.03 (1.02-1.04) | 1.03 (1.02-1.04) |
| Categories of uACR |  |  |
| < 0.3 g/g | Ref | Ref |
| [0.3 - 0.5[ g/g | 1.56 (1.14-2.14) | 1.55 (1.13-2.13) |
| [0.5 - 1.0[ g/g | 2.04 (1.58-2.64) | 1.99 (1.53-2.58) |
| [1.0 - 1.5[ g/g | 2.82 (2.10-3.78) | 2.75 (2.04-3.7) |
| [1.5 - 2.0[ g/g | 4.21 (3.08-5.74) | 4.09 (2.99-5.6) |
| ≥ 2.0 g/g | 4.53 (3.36-6.11) | 4.33 (3.19-5.87) |
| **Kidney replacement therapy** |  |  |
| For each 0.1 g/g higher in uACR | 1.03 (1.02-1.04) | 1.03 (1.02-1.04) |
| Categories of uACR |  |  |
| < 0.3 g/g | Ref | Ref |
| [0.3 - 0.5[ g/g | 1.39 (0.94-2.03) | 1.41 (0.96-2.07) |
| [0.5 - 1.0[ g/g | 1.84 (1.35-2.51) | 1.84 (1.34-2.52) |
| [1.0 - 1.5[ g/g | 2.59 (1.84-3.64) | 2.57 (1.82-3.63) |
| [1.5 - 2.0[ g/g | 4.14 (2.89-5.91) | 4.1 (2.86-5.87) |
| ≥ 2.0 g/g | 4.65 (3.28-6.59) | 4.53 (3.19-6.45) |
| **>30% decline in eGFR** |  |  |
| For each 0.1 g/g higher in uACR | 1.02 (1.01-1.03) | 1.02 (1.01-1.03) |
| Categories of uACR |  |  |
| < 0.3 g/g | Ref | Ref |
| [0.3 - 0.5[ g/g | 1.76 (1.26-2.47) | 1.71 (1.22-2.41) |
| [0.5 - 1.0[ g/g | 2.20 (1.66-2.91) | 2.08 (1.57-2.76) |
| [1.0 - 1.5[ g/g | 2.45 (1.78-3.39) | 2.34 (1.69-3.25) |
| [1.5 - 2.0[ g/g | 3.20 (2.25-4.56) | 3.07 (2.15-4.39) |
| ≥ 2.0 g/g | 3.47 (2.47-4.87) | 3.45 (2.45-4.87) |

Cox models were adjusted for age, sex, hypertension, diabetes, history of myocardial infarction, cerebrovascular disease, peripheral artery disease, heart failure, arrhythmia, acute kidney injury, systolic and diastolic blood pressure, eGFR, hemoglobin, CRP, serum albumin, phosphate, renin-angiotensin system inhibitors, mineralocorticoid receptor antagonists, statins, antiplatelet therapy, corticosteroids, immunosuppressive therapy, all-cause hospitalizations and number of out-patient visits.

MAKE: major adverse kidney events (a composite of kidney replacement therapy and >30% decline in eGFR).

# Table S7. Characteristics of patients with IgA nephropathy by category of 1-year change in urine albumin-to-creatinine ratio (uACR) (N=785)

|  | **NA, %** | **Overall** | **≥ 2-fold uACR decrease** | **Stable uACR** | **≥ 2-fold uACR increase** |
| --- | --- | --- | --- | --- | --- |
|  |  | **N = 785** | **N = 198** | **N = 450** | **N = 137** |
| ***Demographics and clinical data*** |  |  |  |  |  |
| Gender, Women | 0.0 | 201 (25.6) | 56 (28.3) | 107 (23.8) | 38 (27.7) |
| Age, years [Q1-Q3] | 0.0 | 55.9 [43.7, 67.8] | 52.3 [39.8, 66.2] | 56.8 [46.2, 67.9] | 56.3 [42.7, 68.6] |
| Body mass index, kg/m | 39.0 | 28.4 (5.4) | 28.5 (5.9) | 28.5 (5.4) | 28.1 (5.1) |
| Systolic BP, mmHg | 12.1 | 133.8 (15.9) | 130.5 (15.7) | 134.8 (16.0) | 135.1 (15.2) |
| Diastolic BP, mmHg | 12.1 | 80.9 (10.7) | 78.9 (10.1) | 81.1 (10.9) | 82.9 (10.2) |
| ***Comorbidities*** |  |  |  |  |  |
| Hypertension | 0.0 | 667 (85.0) | 165 (83.3) | 388 (86.2) | 114 (83.2) |
| Diabetes mellitus | 0.0 | 106 (13.5) | 28 (14.1) | 62 (13.8) | 16 (11.7) |
| Any cardiovascular disease | 0.0 | 97 (12.4) | 20 (10.1) | 61 (13.6) | 16 (11.7) |
| Myocardial infarction | 0.0 | 42 (5.4) | 8 (4.0) | 25 (5.6) | 9 (6.6) |
| Peripheral vascular disease | 0.0 | 12 (1.5) | 1 (0.5) | 9 (2.0) | 2 (1.5) |
| Cerebrovascular disease | 0.0 | 64 (8.2) | 16 (8.1) | 39 (8.7) | 9 (6.6) |
| Heart failure | 0.0 | 48 (6.1) | 16 (8.1) | 23 (5.1) | 9 (6.6) |
| Arrhythmia | 0.0 | 63 (8.0) | 13 (6.6) | 37 (8.2) | 13 (9.5) |
| Acute kidney injury | 0.0 | 90 (11.5) | 33 (16.7) | 43 (9.6) | 14 (10.2) |
| ***Biological values*** |  |  |  |  |  |
| Hemoglobin, g/dL | 1.7 | 12.6 (1.7) | 12.4 (1.6) | 12.7 (1.7) | 12.6 (1.7) |
| C-reactive protein, mg/L | 28.4 | 5.2 (9.1) | 5.4 (8.9) | 4.7 (7.3) | 6.8 (13.5) |
| Serum albumin, g/L | 3.7 | 36.9 (4.1) | 37.7 (4.1) | 37.0 (3.9) | 35.5 (4.7) |
| eGFR, mL/min per 1.73 m² | 0.3 | 30.9 (19.5) | 33.2 (21.6) | 29.6 (18.3) | 32.2 (19.8) |
| ACR, g/g [Q1-Q3] | 0.0 | 0.5 [0.2, 1.2] | 0.2 [0.1, 0.5] | 0.7 [0.3, 1.3] | 0.9 [0.2, 1.9] |
| ACR, KDIGO categories | 0.0 |  |  |  |  |
| A1: < 30 mg/g |  | 59 (7.5) | 34 (17.2) | 23 (5.1) | 2 (1.5) |
| A2 : 30-300 mg/g |  | 200 (25.5) | 78 (39.4) | 88 (19.6) | 34 (24.8) |
| A3 : > 300 mg/g |  | 526 (67.0) | 86 (43.4) | 339 (75.3) | 101 (73.7) |
| ***Ongoing medications*** |  |  |  |  |  |
| Renin-angiotensin system inhibitors | 0.0 | 686 (87.4) | 174 (87.9) | 392 (87.1) | 120 (87.6) |
| Calcium channel blockers | 0.0 | 456 (58.1) | 122 (61.6) | 260 (57.8) | 74 (54.0) |
| Diuretics | 0.0 | 384 (48.9) | 105 (53.0) | 205 (45.6) | 74 (54.0) |
| B-blockers | 0.0 | 371 (47.3) | 102 (51.5) | 203 (45.1) | 66 (48.2) |
| Corticosteroids | 0.0 | 143 (18.2) | 48 (24.2) | 74 (16.4) | 21 (15.3) |
| Oral immunosuppressive therapy | 0.0 | 45 (5.7) | 20 (10.1) | 21 (4.7) | 4 (2.9) |
| Antiplatelet | 0.0 | 104 (13.2) | 26 (13.1) | 62 (13.8) | 16 (11.7) |
| Lipid-lowering therapy | 0.0 | 413 (52.6) | 92 (46.5) | 243 (54.0) | 78 (56.9) |
| ***Healthcare use in the year prior*** |  |  |  |  |  |
| No. of hospitalizations, median [Q1-Q3] | 0.0 | 0.0 [0.0, 1.0] | 0.0 [0.0, 1.0] | 0.0 [0.0, 1.0] | 0.0 [0.0, 0.0] |
| A least one hospitalization, n (%) | 0.0 | 237 (30.2) | 83 (41.9) | 129 (28.7) | 25 (18.2) |
| At least one CV-related hospitalization, n (%) | 0.0 | 22 (2.8) | 9 (4.5) | 13 (2.9) | 0 (0.0) |
| No. of outpatient visits [Q1-Q3] | 0.0 | 3.0 [2.0, 6.0] | 4.0 [2.0, 7.0] | 3.0 [2.0, 6.0] | 3.0 [1.0, 5.0] |

Categorical variables are reported as frequencies (and percentages), and continuous variables are reported as mean (SD) or median [Q1-Q3: first and third quartile].

# Figure S1. Study design.

**Exclusion: Age < 18 years, non-IgA nephropathy,**

**no ACR measurement**

**Dataset calendar period coverage**

**[2005-01-01 to 2021-12-31]**

**Cohort Entry Date**

**Index date**

**Study time**

**Exclusion: secondary IgAN (see below)**

**Exclusion: maintenance dialysis, kidney transplantation**

**[-∞ ; 0]**

**Baseline covariates: demographics [0]**

**Baseline covariates: comorbidities**

**[-∞ ; 0]**

**Baseline covariates: labs values (most recent)**

**[2005-01-01 ; 0]**

**Baseline covariates: medication use [-180 days ; 0]**

**Follow-up window (ICD-10-based outcomes)**

**[0 ; ≤ 2021-12-31]**

This graphical depiction of study design shows the different time windows used to define exposure (albumin-to-creatinine ratio), exclusion criteria, baseline covariates and outcomes.

| *** Patients with the following ERA-PRD codes were excluded from the study:** | |
| --- | --- |
| **ERA-PRD code** | **Definition** |
| 1137 | Familial IgA nephropathy - no histology |
| 1144 | Familial IgA nephropathy - histologically proven |
| 1159 | IgA nephropathy secondary to liver cirrhosis - no histology |
| 1163 | IgA nephropathy secondary to liver cirrhosis - histologically proven |
| 1504 | Henoch-Schönlein purpura / nephritis - no histology |
| 1515 | Henoch-Schönlein purpura / nephritis - histologically proven |
| 3572 | Unspecified hematuria and isolated proteinuria |
| 3712 | Unspecified hematuria |

# Figure S2. Restricted cubic splines depicting the rates of major adverse kidney events and its individual components across the continuum of baseline urine albumin-to-creatinine ratio (N=1269).


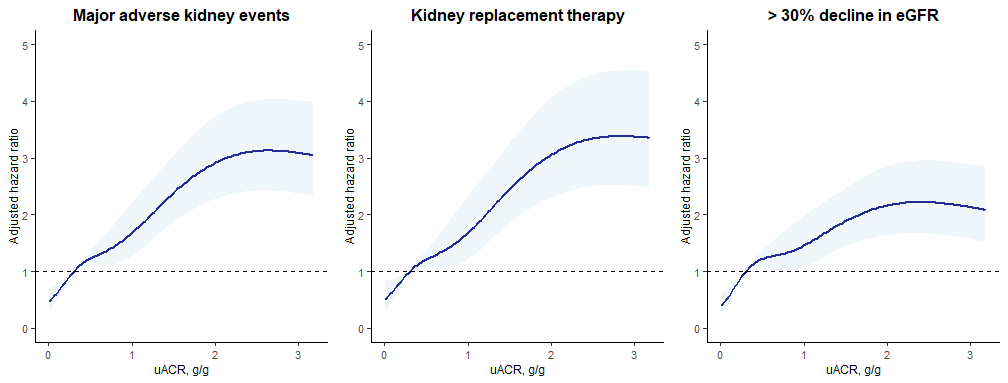


Cox models were adjusted for age, sex, hypertension, diabetes, history of myocardial infarction, cerebrovascular disease, peripheral artery disease, heart failure, arrhythmia, acute kidney injury, systolic and diastolic blood pressure, eGFR, hemoglobin, CRP, serum albumin, phosphate, renin-angiotensin system inhibitors, mineralocorticoid receptor antagonists, statins, antiplatelet therapy, corticosteroids, immunosuppressive therapy, all-cause hospitalizations and number of out-patient visits in the year prior. uACR: urine albumin-to-creatinine ratio.
